# Advancing Inequality Monitoring in Immunization: Reflecting on 10 Years of WHO Contributions

**DOI:** 10.3390/vaccines13101044

**Published:** 2025-10-10

**Authors:** Nicole Bergen, Katherine Kirkby, Anne Schlotheuber, Ahmad Reza Hosseinpoor

**Affiliations:** Department of Data, Digital Health, Analytics and AI, World Health Organization, 20 Avenue Appia, CH-1211 Geneva 27, Switzerland

**Keywords:** capacity strengthening, childhood vaccination, disaggregated data, global analyses, health equity, immunization coverage, inequality monitoring, knowledge translation, reporting, World Health Organization

## Abstract

Major immunization programs and initiatives have prioritized the advancement of equity in immunization. Over the past decade, the World Health Organization has made contributions to understanding inequalities in immunization, including global analyses of immunization inequality as well as tools for knowledge dissemination and capacity strengthening. This article provides an overview of these contributions, highlighting key findings of scholarly reports and journal articles and identifying areas for further research and development to expand monitoring efforts and enhance their impact. Global analyses have primarily drawn from household survey data to explore inequalities related to economic status, education, gender, and geography. Reports and articles address childhood immunization, COVID-19 vaccine indicators, and maternal tetanus protection. Inequalities were reported across all dimensions, with variation by country and income grouping. Time trends generally suggest persistent, though narrowing, inequalities. Areas for further development include the following: increasing awareness and political support for advancing equity in immunization; expanding the collection, availability, and use of disaggregated immunization data; continuous capacity building of inequality monitoring, especially at national and subnational levels; adapting inequality monitoring practices to changing contexts and priorities; strengthening the links between data/evidence and action/impact; and building on existing partnerships and collaborations.

## 1. Introduction

Characterizing and addressing inequalities in immunization is a shared priority of the World Health Organization (WHO), Gavi, the Vaccine Alliance (Gavi), the United Nations Children’s Fund (UNICEF), and other global health and development partners. Over the past decades, the reduction in unfair inequalities has been featured prominently in high-level immunization initiatives, including the Global Immunization Vision and Strategy, the Decade of Vaccines, and the Global Vaccine Action Plan, and, more recently, the Immunization Agenda 2030 (Box 1). During the COVID-19 pandemic, 190 economies signaled alignment with the advancement of immunization equity by signing onto COVID-19 Vaccines Global Access (COVAX), which emphasized the notion that “no one is safe until everyone is safe” [1]. Senior global health experts, academics, and leaders from ministries of health convened the Equity Reference Group for Immunization, raising key challenges and recommendations for improving equity in immunization (these include calls to improve data quality; develop effective vaccine service delivery models; reach underserved children; and bring attention to gender as a cross-cutting and influencing factor [2]) [3].

Box 1 **Global immunization initiatives in the 2000s.**

**Figure 1:**
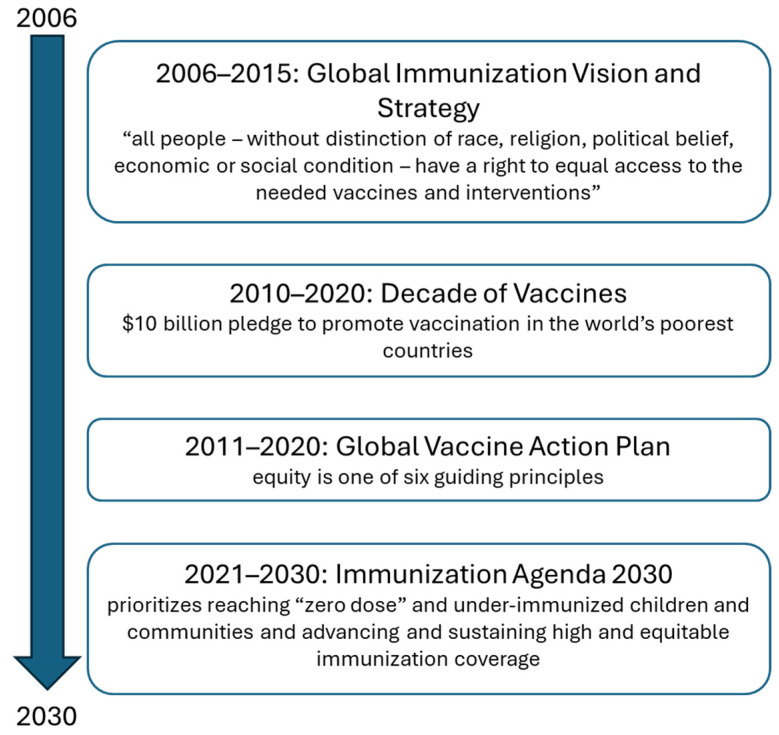
Major global immunization initiatives with a focus on equity.

A series of global immunization initiatives since the early 2000s have highlighted equity in different ways (Figure 1). The Global Immunization Vision and Strategy, 2006–2015, was the first 10-year concerted global plan aiming to realize the potential of immunization [4].

The Decade of Vaccines, 2011–2020, was launched at the 2010 World Economic Forum in Davos. It ushed in the development of the Global Vaccine Action Plan (GVAP), 2011–2020, endorsed by the World Health Assembly in 2012 [5].

The implementation of GVAP and its equity goals required country-level contextualization and uptake via national immunization plans, which occurred only to a limited extent [6]. The inadequate national uptake of GVAP aspirations was partially addressed via Regional Vaccine Action Plans, which helped to support and strengthen national immunization programs. While GVAP’s monitoring and evaluation framework brought attention to gaps in immunization coverage, a focus on global averages masked variations across regions, countries, and subnational areas [6].

In response to lessons learned from GVAP, the Immunization Agenda 2030 (IA2030), 2021–2030, co-created by countries and development partners and endorsed by the World Health Assembly, adopted a data-guided focus, emphasizing country ownership [7]. IA2030 envisions a world where everyone, everywhere, at every age fully benefits from vaccines for good health and well-being.

Health inequality monitoring, which entails the repeated measurement of health differences between population subgroups [8], plays a key role in advancing immunization equity. Immunization equity underscores that everyone “achieve their full health potential regardless of demographic, social, economic, or geographic strata” [9]. Quantifying inequalities in immunization helps to identify population groups that are missing out on the benefits of vaccines and can serve as one form of evidence to help inform equity-oriented policies, programs, and practices.

Over the past decade and in collaboration with global health partners, the WHO has spearheaded a range of initiatives to advance inequality monitoring in immunization. This article provides an overview of these contributions, which encompass global analyses and reports, as well as capacity strengthening resources, data tools, and knowledge dissemination activities. It demonstrates key findings about the state of inequality in immunization and proposes areas for further action to monitor and advance equity in immunization. This article is intended to encourage discussion about the future of measuring inequalities—and advancing equity—in immunization. We also hope it will encourage new research in the topic area.

## 2. Global Analyses of Immunization Inequality

The WHO’s analyses of inequality in immunization have spanned several immunization topics and explored inequalities according to numerous dimensions, including economic status, education, gender, subnational region, and place of residence. Most of these analyses are drawn from household health survey data or administrative data. Review articles have assessed trends in the literature. The use of multiple reporting outputs, from global reports to peer-reviewed publications, has enabled engagement with diverse policymaking, academic, and public health audiences. Detailed information about each analysis, including the methods, findings, implications, and limitations, can be found in the original sources cited throughout Section 2.1 and Section 2.2

### 2.1. WHO Reports

Two scholarly reports, published by the WHO in 2016 and 2018, were dedicated to inequalities in childhood immunization. Drawing on data from 69 low- and middle-income countries, *State of Inequality: Childhood Immunization* assessed inequalities in five childhood immunization indicators, disaggregated by four dimensions of inequality [10]. It concluded that, although inequalities had generally narrowed across the study countries, concerning levels of inequality persisted, especially in relation to household economic status and mother’s education level. For example, one in three countries reported very high levels of absolute economic-related inequality (i.e., coverage at least 20 percentage points higher in the richest than the poorest quintile) for three doses of diphtheria–tetanus–pertussis (DTP3), measles, and full immunization coverage [10]. The follow-up report *Explorations of Inequality: Childhood Immunization* provided a more detailed analysis of the current status of inequality in 10 priority countries [11]. It used multiple regression analysis to identify factors associated with immunization coverage and demonstrated how the likelihood of vaccination is affected by compounded advantage or vulnerability. One finding that emerged from this analysis was the strong impact of accounting for multiple characteristics simultaneously. In Nigeria in 2013, for example, children of mothers aged 20–34 years who were highly educated and living in a rich household in the South South region were 300 times more likely to be vaccinated with DTP3 than children with teenaged mothers with no education, living in poor households in the North West region [11]. Both reports were accompanied by electronic interactive visuals, facilitating further exploration of the data. Table 1 contains an overview of the characteristics of these reports.

The WHO’s flagship report *World Health Statistics 2025: Monitoring Health for the SDGs*, *Sustainable Development Goals* features a chapter on the importance of vaccine equity [12]. The chapter provides examples of inequalities in diphtheria–tetanus–pertussis (DTP) and tetanus immunization coverage across World Bank country income groupings. It highlights contemporary barriers to immunization and data availability issues.

### 2.2. Publications in Peer-Reviewed Journals

Economic-related inequality was assessed in tetanus vaccination coverage in pregnancy [13], non-receipt of DTP immunization in one-year-old children [14], and DTP immunization coverage in one-year-old children [15]. These studies all focused on low- and middle-income countries, drawing data from Demographic Health Surveys (DHS) and Multiple Indicator Cluster Surveys (MICS) and categorizing economic status as five wealth quintiles. Across these studies, immunization inequality overwhelmingly favored the wealthiest quintile. Some key findings from these studies are presented in Box 2.

Box 2 **Key findings from analyses of economic-related inequality in immunization.**

**Johns et al. (2024): Comparison of Wealth-Related Inequality in Tetanus Vaccination Coverage before and during Pregnancy: A Cross-Sectional Analysis of 72 Low- and Middle-Income Countries [13]**

Economic-related inequality in tetanus immunization coverage was calculated using the slope index of inequality and relative index of inequality, which take into account the levels of coverage across the whole economic spectrum. Across the 72 low- and middle-income study countries, absolute inequality in tetanus immunization cov-erage before pregnancy averaged 2.4 percentage points [95% CI 1.0–3.7]; inequality during pregnancy averaged 10.5 percentage points [95% CI 5.3–15.6], and inequality at birth averaged 12.8 percentage points [95% CI 7.7–17.9].

**Bergen et al. (2022): Economic-Related Inequalities in Zero-Dose Children: A Study of Non-Receipt of Diphtheria–Tetanus–Pertussis Immunization Using Household Health Survey Data from 89 Low- and Middle-Income Countries [14]**

Economic-related inequality in the prevalence of zero-dose children was assessed using difference, ratio, slope index of inequality, concentration index, and excess change measures. Within-country economic-related inequalities tended to favor the richest quintile, with 19 of 89 countries reporting a difference of 20.0 percentage points or more between the richest and poorest wealth quintiles. The difference between the median prevalence in the poorest and richest quintiles was higher in low-income countries (14.4 percentage points) than in lower-middle-income countries (8.9 per-centage points) and upper-middle-income countries (2.7 percentage points).

**Hosseinpoor et al. (2016): State of inequality in Diphtheria–Tetanus–Pertussis Immunisation Coverage in Low-Income And Middle-Income Countries: A Multicountry Study of Household Health Surveys [15]**

Economic-related inequality in DTP3 immunization coverage was calculated as difference and ratio measures based on coverage in the richest and poorest wealth quintiles. Within countries, the gap in DTP3 immunization coverage exceeded 20 per-centage points in 20 of the 51 low- and middle-income study countries, with 5 coun-tries reporting a gap of more than 40 percentage points. Time trend analyses in 21 countries showed most countries reporting faster improvements in the poorest quintile than the richest over the previous decade, alongside overall national gains in DTP3 coverage.

Refer to the full publications for more information about their respective methods and results [13,14,15].

Education was explored as a dimension of inequality in an analysis of COVID-19 vaccine indicators in 90 countries [16]. The study described inequalities at the global level, across country-income groups, and at national levels using data from a large multicountry online survey, the University of Maryland Social Data Science Center Global COVID-19 Trends and Impact Survey. It found that, while vaccine receipt tended to be higher among the most educated respondents, patterns of education-related inequality in structural barriers, vaccine hesitancy, and vaccine refusal were more varied. For instance, among unvaccinated people, vaccine hesitancy was higher among those with lower education and vaccine refusal was higher among those with higher education, especially in high-income countries.

A series of analyses investigated gender as a dimension of inequality in childhood immunization [17,18,19]. An analysis of over 160 countries assessed the association between DTP vaccine indicators and gender inequality at the national level [17]. It established that higher levels of national gender inequality were consistently associated with both lower levels of DTP3 immunization and higher levels of non-receipt of DTP. Within-country inequalities related to gender were evident in a study of 52 low- and middle-income countries [18]. Using the Survey-based Women’s emPowERment (SWPER) Global Index (see Box 3) derived from DHS data, DTP immunization indicators were compared across social independence tertiles. Greater social independence among women was associated with better childhood immunization outcomes. The relationship between gender inequality and childhood immunization was further explored through an analysis at the subnational level in 57 countries [19]. The study quantified the associations between the subnational gender development index and DTP immunization indicators, finding that the quintile of subnational regions with higher gender inequality (favoring men) showed lower DTP3 immunization coverage than regions with lower gender inequality.

Box 3 **Survey-based Women’s emPowERment (SWPER) Global Index.**

SWPER is composed of three domains. Social independence scores include variables related to women’s educational attainment, age at pivotal life events, spousal asset differentials and access to information. Decision-making autonomy reflects the degree to which women are involved in household decisions (including household purchases, health care and visiting relatives). The attitude to the violence domain reflects women’s incorporation of gender norms-related acceptability of intimate partner violence.

A separate analysis explored gender-related barriers relevant to maternal tetanus protection in 39 low- and middle-income countries [20]. The study assessed the association between maternal tetanus immunization coverage and SWPER domains as well as other gender-related variables. Women reporting higher SWPER scores for the social independence and decision-making autonomy domains had higher odds of maternal tetanus protection; however, the attitude to violence domain showed no association with maternal tetanus protection.

Patterns in immunization by geographical dimensions have revealed the extent to which national averages conceal inequality. A study of 24 African countries used administrative data to quantify inequalities in DTP1-DTP3 immunization drop-out at the subnational level, noting substantial differences between the best- and worst-performing regions [21]. A further assessment within Malawi addressed the link between childhood immunization coverage and proximity to health facilities in rural settings, drawing from data collected between 2013 and 2016 [22]. For rural-residing children, proximity to a health facility was associated with the likelihood of receipt for some, but not all, vaccines. When adjusted for confounding variables, children residing within 5 km of a vaccine-providing facility were more likely to have received rotavirus vaccine (adjusted odds ratio: 1.63, 95% CI: 1.13–2.33) and measles vaccine (adjusted odds ratio: 1.62, 95% CI: 1.11–2.37); BCG, oral polio vaccine, pentavalent vaccine, and pneumococcal conjugate vaccine 13-valent indicators showed no significant associations [22].

A study of inequalities in maternal and neonatal tetanus immunization across 76 low- and middle-income countries considered four dimensions of inequality: economic status, maternal age, maternal education, and place of residence [23]. In low- and lower-middle-income countries, inequalities were evident for all dimensions; in upper-middle-income countries, inequalities were reported for maternal education and place of residence. The study called for expanded multi-country analyses incorporating a wider range of inequality dimensions, along with more context-specific analyses of selected factors with narrower geographic scope.

Three additional articles overviewed inequality measurement considerations [24] and trends in the published literature [25,26]. Kirkby et al. compared the application of 16 summary measures of health inequality, including absolute and relative versions, for two DTP indicators by three dimensions of inequality (economic status, place of residence, and subnational region) in 92 low- and middle-income countries [24]. While the summary measures generally led to similar conclusions, the study noted that absolute and relative measures sometimes produced differing results. It outlined key considerations related to the selection of simple versus complex measures and weighted versus unweighted measures, as well as sensitivity to outliers, target audience, and interpretation.

Scoping literature reviews addressing inequality in childhood immunization and inequality in COVID-19 immunization provided insights into data sources, study characteristics, analysis approaches, and reporting methods [25,26]. In the case of childhood immunization, studies relied heavily on DHS and MICS data [25]. Economic status and maternal education were the most reported dimensions of inequality, and full vaccination with the recommended vaccine schedule was the most common immunization indicator. Few studies applied summary measures of inequality, with many using the odds ratio resulting from logistic regression models [25]. Inequalities in COVID-19 vaccination were primarily explored in relation to age, race/ethnicity and sex/gender, and articles tended to focus on vaccine initiation, full vaccination and/or receipt of booster [26].

## 3. Knowledge Dissemination and Capacity Strengthening

The WHO has led numerous initiatives to foster knowledge dissemination, strengthen capacity, and develop tools for monitoring inequality in immunization. For three consecutive years (2023–2025), the WHO has led the coordination of and guest edited ‘Inequality in immunization’ special issues in the academic journal *Vaccines*, along with guest editors from Gavi, UNICEF, and the United States Centers for Disease Control and Prevention. In 2023, the special issue focused on characterizing situations of inequality, drivers of inequality, and actions and initiatives that hold promise for enhancing equity of immunization across countries [27]. The 2024 and 2025 editions expand this body of evidence through research and review articles that deepen understandings of immunization inequalities and entry points or modalities to address them in meaningful and lasting ways [28,29]. In collaboration with guest editors, the WHO has hosted launch events to enhance the visibility and impact of these collections. Global webinars showcase research findings and provide a forum for reflections from the guest editors and discussion among members of the wider immunization and public health community [30,31].

**Figure 2:**
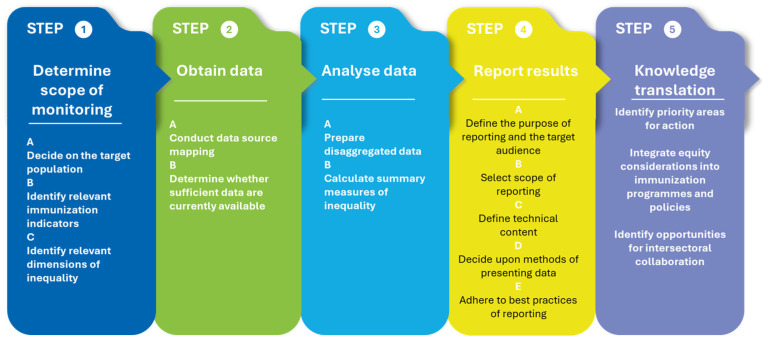
Inequality monitoring in immunization, represented as five steps. Reproduced from [32].

The WHO’s *Inequality Monitoring in Immunization: A Step-By-Step Manual* is a resource to strengthen capacity for the uptake and improvement of inequality monitoring practices in immunization [32]. It is organized in five sections, demonstrating the application of the five steps of inequality monitoring to immunization (Figure 2). Step-by-step guidance, including key questions and best practices for each substep, are summarized in a flow chart, with accompanying examples [32].

As a complement to the step-by-step manual, the WHO eLearning course ‘Inequality Monitoring in Immunization’ was developed to support the skill development of monitoring and evaluation officers, data analysts, academics and researchers, public health professionals, medical and public health students, and others with a general interest in health data, inequality monitoring, and data analysis [33]. The publicly available, self-paced course consists of five learning modules, which introduce the general steps of inequality monitoring and demonstrate their applicability to immunization programs.

The WHO’s Health Inequality Data Repository, which is the largest collection of disaggregated health data, aims to increase the accessibility of data for monitoring inequalities across diverse health topics [34]. It contains datasets about childhood immunization (disaggregated by age, economic status, education, place of residence, sex, and subnational region) and subnational DTP immunization dropout rates (disaggregated by district quintiles), as well as data about COVID-19 vaccination (disaggregated by age, education, gender, health worker status, and place of residence). The WHO Health Equity Assessment Toolkit (HEAT) is a software application that facilitates the exploration, analysis, and reporting of these data. HEAT allows users to assess disaggregated data and summary measures of health inequality, and to create interactive graphs, maps, and tables [35].

## 4. Areas for Further Development

Looking ahead to the next decade and beyond, we highlight several opportunities to advance understandings of inequalities in immunization and progress towards greater equity. Critically, there is a need for sustained and increased awareness and political support for achieving equity in immunization. Although reductions in immunization inequalities have been observed, substantial gaps persist, and continued progress is not assured. Equity should be at the forefront of immunization initiatives and policies across global, national, and subnational levels of influence. For instance, global health agencies, such as WHO, Gavi, and UNICEF, and countries can ensure equity indicators are part of immunization strategies and programs. Prioritizing equity in this manner establishes a mandate for inequality monitoring and its integration into monitoring and evaluation frameworks. Further, it can lead to greater resource allocation for equity-focused monitoring and follow-on actions.

Disaggregated data are a key requirement of inequality monitoring, and their collection, availability, and use are important for identifying and characterizing patterns of inequality. Recent disruptions and threats to the continuity of the DHS program, which is a primary source of data for monitoring inequalities in immunization in many low- and middle-income countries, highlight the need to revisit the administrative volatility of large data collection systems and to ensure that replacement systems are established to preserve scientific rigor and long-term sustainability [36]. Efforts are warranted to improve the quality of disaggregated immunization data and expand the collection of data to encompass a wider range of populations, dimensions of inequality, and immunization-related indicators.

Continued capacity strengthening is needed to promote the analysis and use of such data, especially at national and subnational levels. To this end, the recent establishment of the Health Inequality Monitoring Network, a WHO-led initiative to promote health inequality monitoring, presents a promising opportunity for expanding the reach of capacity-strengthening activities [37]. The WHO’s *Health Inequality Monitoring Atlas* will map Member States’ capacities, policies, and resources for health inequality monitoring, enabling the provision of more targeted national support. Forthcoming WHO-blended learning courses for inequality monitoring in immunization will help to strengthen general approaches to applied inequality monitoring as well as skill development in the use of statistical software programs and packages.

Inequality monitoring practices should adapt to changing contexts and priorities relevant to immunization, and support is needed to do so. For example, Gavi and JSI have been instrumental in establishing Zero-Dose Learning Hubs to identify and reduce inequities that affect children who are unimmunized or under-immunized [38]. In recent years, the WHO published guidance focused on the behavioral and social drivers of vaccination to better understand the drivers of vaccine uptake [39]. Protocols have been developed for the use of geospatial technology for equity-oriented immunization program microplanning activities, such as the use of geographic information systems (GIS) for the collection, management, analysis, modeling, and visualization of geographic data [40].

Further opportunities lie in strengthening the links between inequality data/evidence and action/impact. Effective communication about immunization data can support informed decision-making by a range of stakeholders—from those involved in high-level resource allocation to parents making vaccination decisions for their children [41]. There are promising examples of the use of technologies to deploy evidence-based interventions to strengthen the uptake of vaccines. For instance, UNICEF’s Cranky Uncle mobile game application was developed to combat vaccine misinformation, targeting end users in different communities and countries through locally relevant tailored versions of the game [42].

More broadly, strengthened efforts to monitor and address inequalities in immunization entail building on existing partnerships and forging new collaborations with diverse stakeholders and groups supporting the achievement of immunization targets and goals. The 74th World Health Assembly brought renewed attention to the importance of cross-sectoral strategies, policies, and plans to monitor inequalities and achieve health equity, noting the necessity of inclusive engagement with stakeholders, including intergovernmental and non-governmental organizations and the academic and private sectors [43]. Strong, long-term collaborations are positioned to increase accountability for equity and promote the wider participation of society in advancing equity objectives.

## 5. Conclusions

Over the past decade, the WHO has made a variety of contributions to further inequality monitoring in immunization, upholding the equity-promoting focus of major global immunization initiatives. These contributions have taken the form of global analyses and reports, capacity strengthening resources, research and knowledge dissemination activities, and tools supporting data accessibility, analysis, and reporting. These complementary efforts have been part of a wider movement at the global level to use data and evidence to inform equity-oriented action for immunization.

However, a shifting global health landscape is emerging, characterized by resource constraints, program disruptions, technological changes, health misinformation, and new health threats. Leading up to the launch of Gavi’s new five-year strategy for 2026–2030, Gavi 6.0, and at the midpoint of IA2030, renewed attention is needed to protect and advance work to achieve equity in immunization. This includes the continuance of efforts to collect disaggregated data and mainstream inequality monitoring in immunization programs.

## Figures and Tables

**Table 1:** Characteristics of WHO reports on inequality in childhood immunization.

	State of Inequality: Childhood Immunization	Explorations of Inequality: Childhood Immunization
Year of publication	2016	2018
Study countries	69 low- and middle-income countries	10 priority countries, identified by Gavi, the Vaccine Alliance
Childhood immunization indicators	BCG immunization Measles immunization DTP3 immunization Polio immunization Full immunization	DTP3 immunization
Dimensions of inequality	Household economic status Mother’s education Place of residence Sex	Child’s sex Birth order Household economic status Mother’s age at birth Mother’s education Mother’s ethnicity or caste/tribe Place of residence Sex of household head Subnational regions
Data sources and years	DHS and MICS 1994–2014	DHS 2012–2016
Analysis approach	Descriptive analysis of disaggregated data (latest situation and change over time)	Descriptive analysis of disaggregated data (latest situation) Multiple regression analysis

BCG: Bacille Calmette–Guérin; DHS: Demographic and Health Surveys; DTP3: three doses of diphtheria–tetanus–pertussis vaccine; MICS: Multiple Indicator Cluster Surveys. Refer to the full reports for more information about their respective methods and results [10,11].

## Data Availability

The reports, studies, tools, and resources cited in this article are all available in the public domain.

## Abbreviations

The following abbreviations are used in this manuscript:

BCG	Bacille Calmette–Guérin
COVAX	COVID-19 Vaccines Global Access
DHS	Demographic and Health Surveys
DTP	Diphtheria–tetanus–pertussis
DTP1	One dose of diphtheria–tetanus–pertussis vaccine
DTP3	Three doses of diphtheria–tetanus–pertussis vaccine
Gavi	Gavi, the Vaccine Alliance
GVAP	Global Vaccine Action Plan
HEAT	Health Equity Assessment Toolkit
IA2030	Immunization Agenda 2030
MICS	Multiple Indicator Cluster Surveys
SWPER	Survey-based Women’s empowerment
UNICEF	United Nations Children’s Fund
WHO	World Health Organization
